# Dynamic Cholesterol-Conditioned Dimerization of the G Protein Coupled Chemokine Receptor Type 4

**DOI:** 10.1371/journal.pcbi.1005169

**Published:** 2016-11-03

**Authors:** Kristyna Pluhackova, Stefan Gahbauer, Franziska Kranz, Tsjerk A. Wassenaar, Rainer A. Böckmann

**Affiliations:** 1 Computational Biology, Department of Biology, Friedrich-Alexander University Erlangen-Nürnberg, Erlangen, Germany; 2 Computer Graphics, Department of Computer Science, Friedrich-Alexander University Erlangen-Nürnberg, Erlangen, Germany; 3 Groningen Biomolecular Sciences and Biotechnology and Zernike Institute of Advanced Materials, University of Groningen, The Netherlands; University of Virginia, UNITED STATES

## Abstract

G protein coupled receptors (GPCRs) allow for the transmission of signals across biological membranes. For a number of GPCRs, this signaling was shown to be coupled to prior dimerization of the receptor. The chemokine receptor type 4 (CXCR4) was reported before to form dimers and their functionality was shown to depend on membrane cholesterol. Here, we address the dimerization pattern of CXCR4 in pure phospholipid bilayers and in cholesterol-rich membranes. Using ensembles of molecular dynamics simulations, we show that CXCR4 dimerizes promiscuously in phospholipid membranes. Addition of cholesterol dramatically affects the dimerization pattern: cholesterol binding largely abolishes the preferred dimer motif observed for pure phospholipid bilayers formed mainly by transmembrane helices 1 and 7 (TM1/TM5-7) at the dimer interface. In turn, the symmetric TM3,4/TM3,4 interface is enabled first by intercalating cholesterol molecules. These data provide a molecular basis for the modulation of GPCR activity by its lipid environment.

## Author Summary

The G protein coupled C-X-C chemokine receptor type 4 (CXCR4) is a transmembrane receptor that plays an essential role in the human immune system. However, overexpression of CXCR4 has been shown to lead to metastasis of breast and lung cancer cells. Furthermore, the HI-Virus infects T-cells through binding to CXCR4 resulting in immunodeficiency and AIDS. The function of CXCR4 as well as its interaction with HIV depends on the presence of cholesterol in the cell membrane. Additionally, dimerization of CXCR4 is considered to be functionally relevant. Here, using more than a thousand of molecular dynamics simulations, we aim to connect the cholesterol-modulated function of CXCR4 with a potentially cholesterol-conditioned dimerization. Our results show that the dimerization pattern of CXCR4 is drastically altered by cholesterol. Further, we suggest that a functionally competent CXCR4 dimer is exclusively formed in the presence of cholesterol, while the preferred CXCR4 dimer interface in the absence of cholesterol is shifted to probably inactive forms.

## Introduction

Signaling across cell barriers by G protein coupled receptors (GPCRs) is increasingly associated with GPCR dimerization or more general oligomerization [1]. While G protein coupling was shown for many GPCRs to not necessarily depend on dimerization, a direct coupling of function with homo- or hetero-dimerization was reported for a number of GPCRs from both class A and class C [2–4].

The chemokine receptor type 4 (CXCR4) is supposed to form dimers for proper biological activity [5]. This class A GPCR is responsible for directed migration of cells (chemotaxis) in nerve and immune cells of the human body [6, 7]. The overexpression of CXCR4 has been shown to lead to metastasis [8] and increased levels of CXCR4 expression were in particular found in breast and lung cancer cells [9]. Moreover, CXCR4 is known to be a major receptor for the human immunodeficiency virus-1 (HIV-1) upon infecting human T-cells leading to immunodeficiency and AIDS [10]. CXCR4 dimers have been suggested to be either preformed, induced, or stabilized by the interaction with its agonist CXCL12 [5, 11–13].

Function of CXCR4 requires the presence of cholesterol [14]. Cholesterol may influence CXCR4 function by either inducing conformational changes upon binding or by cholesterol-altered CXCR4 dimerization. So far, no direct proofs for either of those mechanisms exist. For cancer cells, the chemotaxis response following CXCL12 binding to CXCR4 was abolished, inhibiting CXCR4-mediated cell migration, if the receptor homodimerization was modulated by removing cholesterol or blocked by a TM4 peptide (an analog of the transmembrane (TM) helix 4 of CXCR4) [14, 15]. Another study revealed that the CXCL12-induced increase of the bioluminescence resonance energy transfer (BRET)-signal intensity, was greatly reduced in presence of TM4 peptides in living cells, whereas TM6 and TM7 peptides showed significantly weaker influence [16]. In addition, CXCR4-mediated inhibition of cAMP production was almost abolished due to the binding of TM4 peptides. These observations hint to a mechanism where dimeric interfaces involving TM4 enable ligand-induced conformational changes which are required for CXCR4 signaling.

Moreover, the dimerization of wildtype CXCR4 with a truncated form was shown to cause the WHIM (Warts, Hypogammaglobulinemia, Infections and Myelokathexis) syndrome [17].

**Fig 1:**
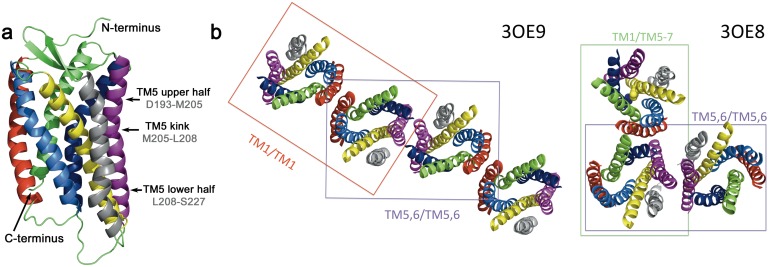
Crystal structures and dimers of CXCR4. (**a**) Structure of CXCR4 in cartoon representation. The coloring of the TM is as follows: TM1 is shown in red (Ala34-Gly64), TM2 in marine blue (Met72-Ala100), TM3 in yellow (Asn106-Val139), TM4 in grey (Gln145-Phe174), TM5 in magenta (Asp193-Ser227), TM6 in dark blue (Leu238-Leu267), and TM7 in green (Cys274-Leu301). (**b**) Dimerization interfaces as observed in crystal structures (pdb entries 3OE9 and 3OE8 [17]).

Structural information on GPCR dimerization or oligomerization interfaces mainly comes from available crystal structures of class A GPCRs [18, 19] displaying preferred interfaces under crystal conditions. Different dimer interfaces were found for various receptors: the transmembrane helix 1 (TM1) and helix 8 (H8) form the symmetric dimer interface of opsin [20] and of the human *β*_2_-adrenergic GPCR [21]. TM1 and TM2 as well as H8 were found at the symmetric interface of the human *κ* opioid receptor [22], and TM1,7 and H8 for rhodopsin [23]. For the latter, atomic force microscopy data further hinted to TM4,5 as the physiological interface [24]. For the C-C chemokine receptor type 5 (CCR5) a crystal packing with an asymmetric interface including TM1,7 and H8 on one monomer and TM4,5 for the second was obtained [25]. Some receptors even displayed different dimer configurations in different crystals. E.g., for the *β*_1_-adrenergic GPCR both symmetric TM1,2,H8 as well as TM4,5 interfaces were observed [26]. Also the high-resolution crystal structures of CXCR4 in complex with either a small antagonist (IT1t), a cyclic antagoinst (CVX15), or a viral chemokine antagonist (vMIP-II) showed symmetric TM5,6 interfaces as the dominant dimerization interfaces [17, 27]. However, CXCR4 dimers were likewise formed by TM1/TM5-7 interactions, or displayed TM1/TM1 as a dimerization interface (Fig 1).

Another important source of information about GPCR dimerization are *in silico* molecular dynamics (MD) simulations. Coarse-grained (CG) simulations allow to study GPCR self-assembly on the microsecond timescale. The two commonly used techniques to obtain a statistical view on the oligomerization process include simulations of multiple receptors [28, 29] and multiple independent parallel dimerization or oligomerization simulations [29–31]. These *in silico* methods have shown that individual GPCRs may interact by different interfaces and suggested for the *β*_2_-adrenergic receptor a possible modulation of the dimer interface by cholesterol [31]. The strength of GPCR dimer interfaces may be approximated by analyzing the potential of mean force [32, 33]. This methodology has shown that both the *β*_1_- and *β*_2_-adrenergic receptors prefer the symmetric TM1,H8 dimerization interface over the also symmetric TM3,4 interface [32]. Rhodopsin also mostly favors the TM1,H8 interface, followed closely by symmetric TM5 and TM4,5 interfaces [33].

A coupling between GPCR function and dimer interface configuration was first shown by cross linking experiments. These revealed that the homodimer interface of the dopamine receptor (class A GPCR) differs considerably between active (TM4/TM4) and inactive (TM4,5/TM4,5) states of the receptor [34]. Similarly, the metabotropic glutamate receptor (mGluR, class C GPCR) adopts different dimer configurations depending on whether the receptor is in its active (TM6/TM6) or inactive (TM4,5/TM4,5) state [35]. Differently, the serotonine receptor (5HT2c, class A GPCR) was found to form both TM1/TM1 and TM4,5/TM4,5 dimers, however, only the latter dimer was activation sensitive [36]. While evidence for a coupling of GPCR function and dimerization is increasing, it is not known whether activation selects between different pre-existing GPCR dimer configurations or whether activation drives a reorientation of the receptors in a dimer into an activation-compatible state.

A couple of recent crystal structures revealed intercalating cholesterol molecules within assembled GPCR oligomers. In case of the A_2*A*_ adenosine receptor, three cholesterol molecules from the upper membrane leaflet were reported to intercalate at the TM1-TM3/TM5,6 dimer interface. The cholesterol molecules were bound between TM2 and TM3 and closely packed around TM6 of the protomers [37]. The symmetric TM5,6/TM5,6 dimer interface of the P2Y12 receptor was stabilized by two cholesterol molecules from the lower leaflet which intercalate between TM3 and TM5 of the protomers [38]. Interestingly, the antagonist-bound, (and thus considered inactive) *μ*-opioid receptors shows a symmetric dimer interface involving TM5, TM6 and cholesterol bound to TM6 [39]. Also for the agonist-bound receptor, in orchestra with a G protein mimetic fragment coupled to the intracellular part, a cholesterol molecule bound to TM6. However, the dimerization via symmetric TM5,6 interactions was disabled due to the outwards movement of the lower part of TM6 [40]. On the other hand, the symmetric TM1,2,H8/TM1,2,H8 dimer (also formed in the inactive state) could still be observed. These observations from different GPCR dimer structures further support the hypothesis that GPCR dimer interfaces may differ between active and inactive configurations. Furthermore, cholesterol binding appears to be receptor specific and thus hard to classify in a general way.

Here, we report a combined coarse-grained and atomistic simulation study of the dimerization of the chemokine receptor type 4, showing a promiscuous, however, specific binding pattern of CXCR4 in a phospholipid bilayer. Our study further pinpoints how cholesterol binding to a specific binding site on TM1,7 largely suppresses the formation of a dimer interface involving TM6, which was reported to be essential in receptor activation [41]. In contrast, cholesterol-rich domains are shown to favor a symmetrical TM3,4 dimer interface of CXCR4. These findings connect the experimental observations that CXCR4 signaling is cholesterol-dependent and that the TM4 helix is a key player in building up signaling-competent homodimer interfaces in a molecular manner.

## Results

The dimerization of CXCR4 in absence and presence of cholesterol was studied using ensembles of MD simulations at a coarse-grained level. In total, we performed 1,500 coarse-grained simulations of two CXCR4 monomers embedded in a phospholipid bilayer built of 1-palmitoyl-2-oleoyl-sn-glycero-3-phosphocholine (POPC) molecules at 0%, 10%, and 30% cholesterol content [30]. The receptors were initially separated by at least 3.5 nm, the simulation lengths were 3 *μ*s (0% cholesterol) and 6 *μ*s (10% and 30% cholesterol) for every simulation, accumulating to a total simulation time of 7.5 ms.

### Promiscuous dimerization of CXCR4

Dimerization of CXCR4 in pure POPC was observed in ≈ 50% of all simulations within 3 *μ*s (For a sample dimerization process see S1 Video). Addition of cholesterol led to a markedly decreased dimerization: Association was obtained in 29% of all simulations at 10% cholesterol concentration, and only 10% of the systems dimerized at 30% cholesterol content within 3 *μ*s (145 and 53 simulations resulting in dimer formation, respectively, (see S1 Fig)). After 6 *μ*s of simulation time, 55% and 21% of the simulation systems at 10% and 30% cholesterol content, respectively, showed dimerization. It is interesting to note that the decrease in dimerization can not be attributed to the decrease in protein mobility only: cholesterol reduced the diffusion coefficient of a monomeric receptor only by 25% and 50% (for 10% and 30% cholesterol, respectively), compared to a corresponding decrease in dimerization by 42% and 79% (see Materials and Methods). However, assuming a simple ‘hit-and-dimerize’ model, a reduced diffusion coefficient would lead to a proportionally decreased receptor dimerization. This finding suggests that cholesterol affects dimerization either by changing the membrane properties or by direct interaction with the receptors. For all dimerization simulations, the receptor did not substantially affect the membrane thickness in its surrounding, i.e. the lipid bilayer frustration [42] is expected to be of minor importance for the dimerization in the POPC systems (compare S1 Table). A few dissociation events of CXCR4 dimers could be observed, allowing to estimate a lower bound for the binding free energy between -20 kJ/mol (30% cholesterol) and -24 kJ/mol (pure POPC, see Materials and Methods).

**Fig 2:**
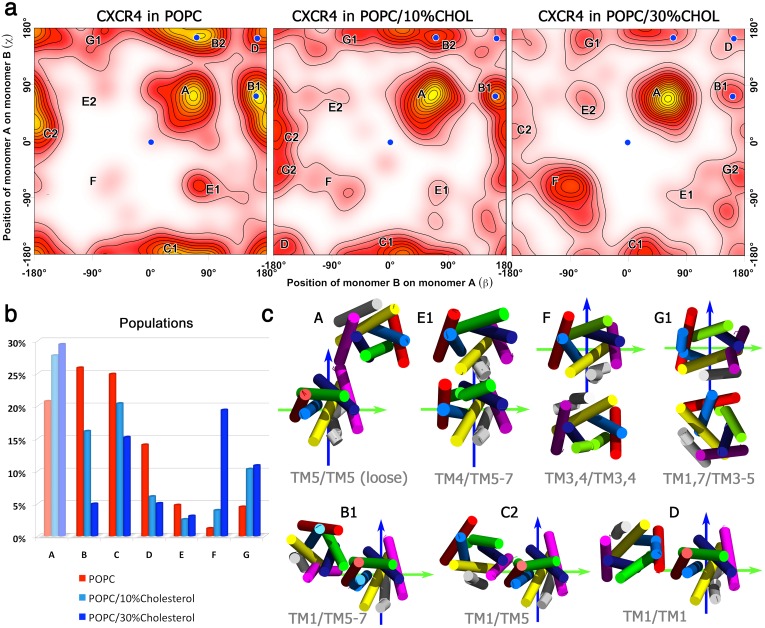
Spontaneously assembled CXCR4 dimers. (**a**) Sampled CXCR4 dimer configurations in pure POPC bilayers after 3 *μ*s (left), and in mixed POPC:cholesterol bilayers after 6 *μ*s simulation time at 10% (middle) and 30% cholesterol content (right). The *β* and *χ* angles are defined relative to the crystal TM5,6/TM5,6 interface (central blue dots, see Methods Section for the exact definition of the angles *β* and *χ*). (**b**) Relative populations for the dimer configurations defined in (a) and (c). (**c**) Representative dimer configurations corresponding to labeled maxima *A* to *G* in (a) are shown in cylindric representation and colored equally as in Fig 1. Symmetric structures (*C1* and *C2* corresponding to TM1/TM5 and TM5/TM1, respectively) are distinguished by indices 1 and 2. The reference structure for all dimer configurations is indicated by green and blue axes.

Overall, an orientation analysis of the monomers in the sampled dimer structures revealed seven different dimer configurations (Fig 2). The most populated dimers in pure POPC were a compact asymmetric TM1/TM5-7 dimer (configuration *B*, population 26%), a TM1/TM5 dimer (*C*, 24%), a loosely bound configuration via terminal TM5/TM5 contacts (*A*, 20%), and a symmetric TM1/TM1 dimer (*D*, 13%). Interconversions between initially formed dimer interfaces were hardly observed with the exception of transitions between interfaces *B* and *C* (compare S2 Table).

**Fig 3:**
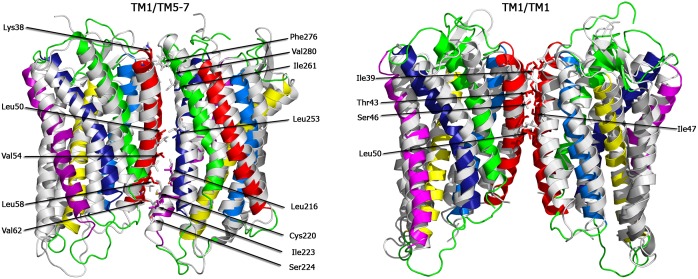
TM1/TM5-7 and TM1/TM1 dimer interfaces and structure alignments. Structure alignments between spontaneously formed TM1/TM5-7 dimer (a) as well as TM1/TM1 dimer (b), obtained from a 3 *μ*s simulation in pure POPC and backmapped to atomistic resolution (colored TM helices), and the crystal structures of TM1/TM5-7 and TM1/TM1 found in PDB-entries 3OE8 and 3OE9, respectively (light grey). The interacting residues are highlighted as sticks and labeled.

The most populated dimer configuration *B* (TM1/TM5-7) is in excellent agreement with one of the available CXCR4 dimer crystal structures (marked as blue spots in Fig 2a, root mean square deviation, RMSD, 3.9 Å, for a molecular view see Fig 3): The crystal structure (pdb entry 3OE8 [17]) has a symmetric TM5,6/TM5,6 interface in a trimeric complex, but also contains an interface formed by TM1 of one monomer and residues of TM5, TM6, and TM7 of the second receptor (Figs 1b and 3a). Specifically, at the N-terminal side of the receptor, Lys38 (TM1) and the aromatic Phe276 form a *π*-H pair and Ile261 (TM6) interacts with the backbone of Lys38. The central part of the interaction interface is built up by hydrophobic interactions between leucines at positions 50 (TM1) and 253 (TM6). Contacts between Val54, Leu58, and Val62 of TM1 and Leu216, Cys220, Ile223, and Ser224 of TM5 complete this dimeric CXCR4 interface at the C-terminal side.

The second most populated dimer configuration (*C*) contains an asymmetric TM1/TM5 interface. A similar interface was reported for the CCR5 chemokine receptor [25], although there TM4 contributed as well to the interface. The symmetric TM1/TM1 interface (observed for 13% of all spontaneously formed dimers in pure POPC, *D*) is also seen in the contact region between two TM5,6/TM5,6 dimers in the crystal structure (compare Fig 1b, pdb entry 3OE9). This dimer interface is mainly built by Thr43 and Ile47 of each receptor (RMSD between crystal and simulated TM1/TM1 amounts to 4.1 Å, Fig 3b).

In contrast to the well reproduced TM1/TM5-7 and TM1/TM1 crystal dimers, the rather compact TM5,6/TM5,6 crystal interface was not sampled in the simulations regardless of the cholesterol concentration. The formation of this dimer interface was found to be hindered by significant differences in the tilt of TM5 between the monomeric CXCR4 form (tilt of upper part of TM5, Asp193-Met205, 28°; lower part of TM5, Val206-Ser227, 35°) and the TM5,6/TM5,6 crystal dimer (tilts of 12° and 22°, respectively), a result that was obtained independent of the membrane thickness (S2 Fig, S1 Table). I.e., under crystallization conditions the TM5 helix in the symmetric TM5,6 dimer is oriented more parallel to the (hypothetical) membrane normal as compared to CXCR4 monomers in a phospholipid membrane. This finding suggests that the formation of the crystal compact TM5,6/TM5,6 dimer in biological membranes requires either prior or induced receptor tilting upon dimerization or conformational receptor rearrangements resulting in an altered TM5 tilt. Additionally, atomistic simulations of the crystal TM5,6/TM5,6 dimer showed an asymmetric orientation of the TM5 helix at the interface, suggesting a reduced stability of the crystal TM5,6/TM5,6 CXCR4 dimer symmetry in a membrane environment (S3 Fig).

A different picture emerges for the TM1/TM5-7 and TM1/TM1 dimer configurations. Here, the TM5 helices of the simulated crystal dimers adopt similar orientations to those observed in CXCR4 monomer simulations.

### Cholesterol-conditioned changes of the CXCR4 dimerization interface

The presence of cholesterol in the lipid bilayer significantly affected the preference for the formation of different dimers. While the population of dimer *A* (loose TM5/TM5 dimer) was only slightly affected, the population of dimers including helices TM1 and TM5-7 (dimers *B*, *C*, and *D*) decreased significantly with increasing cholesterol concentration. At high cholesterol concentration, the formation of dimers *B* and *D* (found in crystal structures) is impeded resulting in relative populations of only ≈ 5%. On the other hand, in the presence of cholesterol dimers interacting via the TM3,4 interface (dimers *F*, *G*) are substantially enhanced. The abundance of the asymmetric dimer *G* (TM1,7/TM3-5 interface) was already enhanced at 10% cholesterol content while the occupancy of the symmetric dimer *F* (TM3,4/TM3,4), hardly sampled in a pure POPC membrane (≈ 1%), increased to ≈ 20% at high cholesterol concentration. These changes in the dimerization profile were largely induced by specific cholesterol-receptor interactions (see below).

#### Cholesterol binding sites on CXCR4

**Fig 4:**
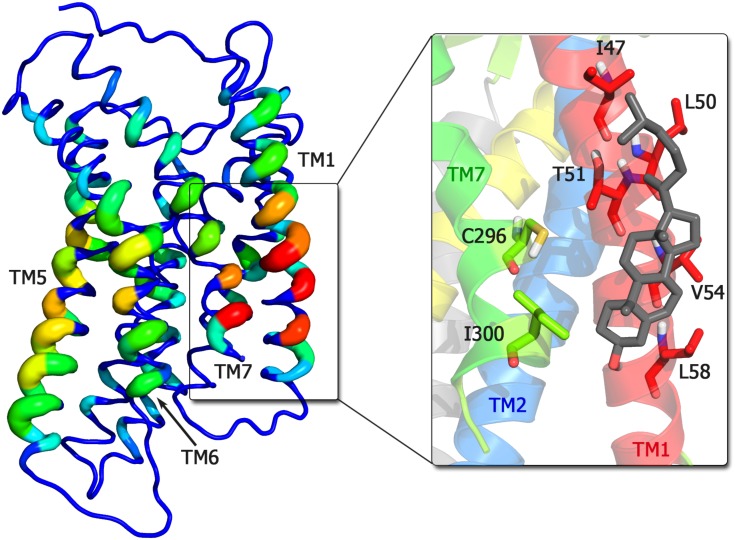
Cholesterol occupation hotspots on CXCR4. Cholesterol binding occupancy in simulations of CXCR4 dimerization at 10% cholesterol concentration. The color and thickness coded residue-resolved occupancy scale ranges from 0 to 65% time occupancy (red, thick). The inset shows a detailed view on the main cholesterol binding site at the TM1,7 interface with the main involved residues in stick representation. Additionally, a bound cholesterol molecule is depicted (gray).

As shown in Fig 4 and in S4 Fig, cholesterol binds to distinct areas on the CXCR4 monomer. The highest cholesterol occupancy, with cholesterol bound for 65% of the simulation time, was observed for a binding spot between helices TM1 and TM7 (shown in detail in the insert of Fig 4 and named lower TM1,7). This binding spot involves residues Ile47, Leu50, Thr51, Val54, Leu58 on TM1, as well as Cys296 and Ile300 on TM7. The average contact time of a bound cholesterol molecule at the TM1,7 binding spot was 6 ns. On the upper part of the TM1 helix another cholesterol binding spot (upper TM1) is located. This spot involves residues Ile39, Pro42, Thr43, Ser46, and Ile47 and the average contact time of bound cholesterol was 5.8 ns. Also the TM5 helix shows strong cholesterol binding as in total three cholesterol binding spots could be identified: The first, upper TM5, consists of Leu194, Val198, Phe201, and Gln202 and cholesterol was on average for 5.1 ns in contact with these residues. The second cholesterol binding spot, termed central TM4,5, is formed by residues Met205, Val206, Ile209, and Leu210 on TM5 as well as by Pro163 and Leu167 on TM4. The average cholesterol contact time for this spot was 2.7 ns, as compared to 4.5 ns for the third binding spot termed lower TM5. The latter cholesterol binding region is formed by Leu216, Ser217, Cys220, Ile221, Ile223, Ser224, and Lys225. Cholesterol binding became more pronounced and less specific for higher cholesterol concentrations (S5 Fig).

Overall, cholesterol binds to the protein mainly via hydrophobic interactions, i.e. hydrophobic residues showed the highest cholesterol occupancies. The spatial distribution functions of cholesterol and its polar headgroup around the receptor revealed that the orientation of bound cholesterol molecules is quite restricted for each binding hot spot (see S4 Fig). Consequently, cholesterol appears to associate with the receptor due to shape-driven interactions allowing the molecules to cover certain parts of the rugged surface of CXCR4.

The specific cholesterol binding spot on TM1,7 is responsible for the reduction of CXCR4 dimerization discussed above via TM1 and TM7 by partial steric blocking of these interaction surfaces.

The reported conserved cholesterol consensus motif (CCM, primarily located on TM4), that was found for the *β*_2_ adrenergic receptor [43], could not be identified. In addition, enhanced binding to possible cholesterol recognition amino acid consensus (CRAC) motifs, as suggested for the serotonin 5-hydroxytryptamine(1A) receptor [44], did not appear in the simulations, eventhough three CRAC motifs could be identified in the sequence of CXCR4. These observations are in agreement with a cholesterol docking study on CXCR4 [45].

#### Cholesterol intercalation stabilizes a symmetric TM3,4/TM3,4 dimer interface of CXCR4

**Fig 5:**
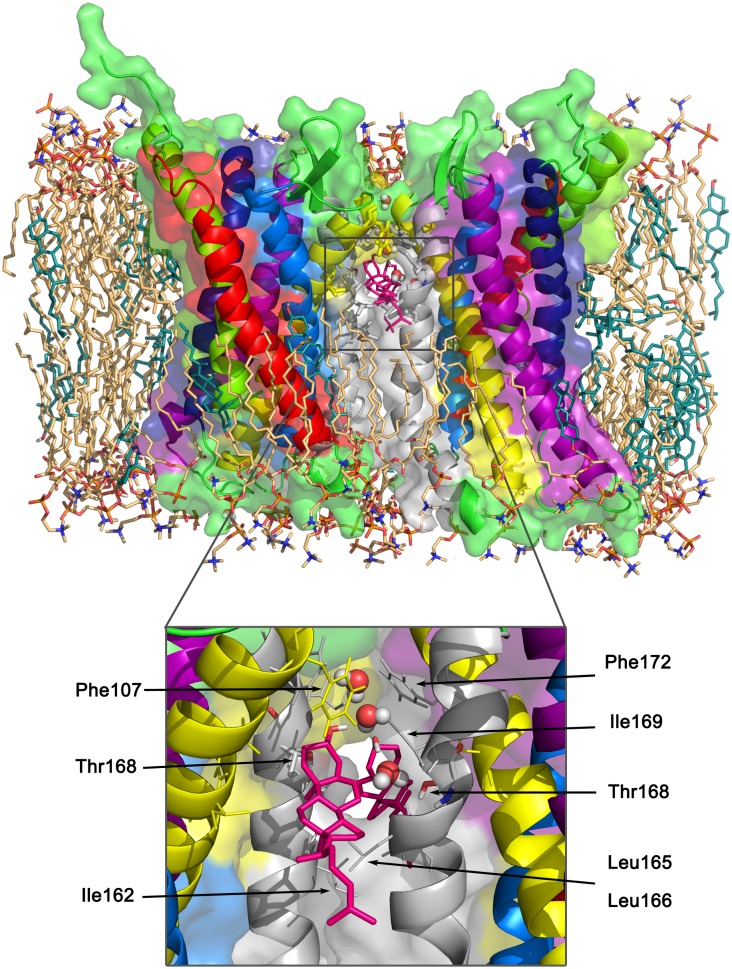
Cholesterol intercalation at the cholesterol-induced dimeric interface. TM3,4/TM3,4 dimer *F* with two symmetrically intercalated cholesterols (shown as hotpink sticks) (structure after 100 ns atomistic MD simulation). The surrounding membrane is shown in light orange (POPC) and teal (cholesterol) sticks. In the insert, the two intercalating cholesterol molecules are highlighted together with their surroundings and water molecules (shown as spheres) forming bridges between the cholesterols and between Thr168 and cholesterol.

Cholesterol additionally temporarily occupied the TM3,4 interface. However, the steroid does not specifically bind at the dimerization interface but rather fills up free volume at this interface. As shown in Fig 5, two symmetrically bound cholesterol molecules intercalate, together with a few water molecules, into the TM3,4 interface (compare also S6 Fig). Phosphocholine lipids can neither fill this space from the top nor from the bottom layer because the cavity is isolated within the bilayer hydrophobic core.

The presence of cholesterol molecules at TM3,4 is responsible for the cholesterol-induced increase of the populations of dimers *G* and *F*. For the former, asymmetric one, only one interaction interface is formed by helices TM3,4. As only one cholesterol molecule is required for this dimer to be formed, even low cholesterol concentrations are sufficient to enhance the formation of dimer *G*. In case of the symmetric dimer *F* (two TM3,4 interfaces) two cholesterol molecules have to be bound simultaneously to smoothen the interaction surface (S2 Video). Therefore, the probability of this compact dimer increases with increasing cholesterol concentration.

## Discussion

The function of several G protein coupled receptors was shown before to be coupled to dimerization. Recent pieces of evidence further prompted to the existence of multiple dimer interfaces, and the selective importance of individual interfaces for signaling. In addition, crystallographic studies suggest that GPCRs may adopt type-specific dimer configurations, despite their structural similarity.

Here, based on ensembles of dimerization simulations, a high plasticity of the dimer interface of the chemokine receptor CXCR4 is reported. Three compact dimer structures emerged as main configurations in a cholesterol-free membrane, involving a TM1/TM5-7 interface, a TM1/TM5 interface, as well as a symmetric TM1/TM1 configuration. Interestingly, the symmetric and compact dimer including helices TM5 and partially TM6, predominant in crystals of the receptor, could not be reproduced in the membrane environment. The reason for this difference could be ascribed to the different orientations of the TM5 helix in the membrane-embedded CXCR4 monomer and the crystal TM5,6/TM5,6 dimer.

In cholesterol-depleted membranes CXCR4 is known to be inactive [15]. GPCR activation and desensitization are connected with movements of TM6 and TM7, respectively [41]. Therefore, in the absence of cholesterol, the involvement of these helices in the dimerization interface, as reported here, is likely to hinder their activation-related movements resulting in chemotaxis-inactive dimers. This interpretation is further supported by a FRET study [15] that reported only a small reduction of FRET signals (15–20%) between CXCR4 proteins upon depletion of cholesterol, while the CXCR4 chemotaxis response to CXCL12 was completely abolished. Either the binding of the CXCL12 ligand (or its dimer) is disabled or the G protein signaling is disrupted.

Addition of cholesterol drastically affected the dimerization pattern: The cholesterol binding between TM1 and TM7 mostly disabled the formation of the TM1/TM5-7 dimer. In turn, a new cholesterol-induced symmetric TM3,4 dimer was enabled. This novel TM3,4 CXCR4 binding mode in cholesterol-rich membranes is corroborated by the experimental finding that addition of TM4 peptides weakened CXCR4 interactions and signaling in malignant cells [15]; the binding of TM4 peptides to CXCR4 likely competes with dimerization at this interface.

Additional experimental support for the functional role of dimerization for signaling is provided by earlier work of Percheranicer *et al.* on HEK cells: Using BRET the authors demonstrated a CXCL12-induced rearrangement of CXCR4 homodimers coupled to the activation of the receptor [16]. TM6 and TM7 peptides did not affect these CXCL12-induced configurational changes, however, addition of these transmembrane peptides significantly lowered the receptor signaling.

Together with our results, this experiment may be interpreted as follows: We assume a pool of different dimers, both active or activatable and inactive, formed by CXCR4 in cholesterol containing cells. Upon addition of CXCL12 the preformed TM3,4 homodimers are stabilized by the interaction with the ligand, shifting the pool towards this dimer configuration and resulting in the observed increased BRET intensity and receptor signaling. Addition of TM4 peptides impedes the formation of this complex resulting in drastically decreased BRET intensity and receptor signaling [15, 16]. In turn, addition of TM6 and TM7 peptides hardly affect the formation of the activation-competent TM3,4 dimer but probably blocked receptor activation by binding to the activation site. Therefore, not the BRET intensity but the activity is influenced by the presence of TM6 or TM7 peptides in the experiment [16]. This in turn supports the conclusion derived above, that dimers including TM6 at the interaction interface likely represent inactive dimers.

Activation-competent CXCR4 dimers are therefore suggested to necessitate binding via the TM3,4 interface which can only be achieved by cholesterol intercalation as shown here.

To further validate these findings, a number of experiments could be performed to analyse different aspects of the discussed results. E.g., cross-linking experiments are highly effective in identifying TM helices that contribute to dimeric interfaces and further to test dimer function. In a similar way as performed by Guo *et al.* [34], cross-linking different sets of substituted cysteines on distinct TM helices will provide experimental insight in how different dimer interfaces can affect agonist-induced activation. Adding agonists to cross-linked TM1/TM5-7 dimers, should result in reduced CXCR4 signaling as compared to adding agonists to cross-linked TM3,4/TM3,4 dimers. In order to address the functionality of cholesterol-induced TM3,4/TM3,4 dimers, mutations of residues on TM3 and TM4 can be introduced that stabilize this interface in the absence of cholesterol, e.g. mutations that insert bulky and hydrophobic residues in order to pad the otherwise cholesterol-filled volume at the interface (e.g. Leu166Phe, Thr168Trp or Ile169Phe). According to our observations, CXCR4 signaling is suggested to be less cholesterol-dependent for these mutants.

In summary, our results suggest a highly dynamic dimerization pattern for the chemokine receptor CXCR4, and provide evidence for a modulation of the dimerization pattern by the membrane composition. In agreement with experiments, the total dimerization is only slightly affected by cholesterol while the specificity of the interaction interface and thus probably the signaling capability are drastically altered.

## Materials and Methods

All dimerization molecular dynamics simulations were prepared using DAFT [30] and performed with GROMACS 4.6.x [46] using the coarse-grained Martini force field [47]. In total, three CXCR4 (Fig 1a) dimerization assays with 0%, 10%, and 30% cholesterol content in a POPC lipid bilayer were studied, comprising ≈ 500 independent simulations each. The simulation lengths were 3 *μ*s each for the pure POPC bilayer systems and 6 *μ*s for the mixed POPC:cholesterol membranes (consisting of POPC:cholesterol in 9:1 ratio and in 7:3 ratio) in order to account for the reduced diffusion and dimerization kinetics of the receptors.

The orientation of CXCR4 monomers in membranes of varying thickness as well as the preferential cholesterol binding sites were addressed in additional simulations at both CG and atomistic resolution. Moreover, for analysis of the dimer interfaces and lipid-protein interactions selected CG structures were converted back to atomistic resolution [48] and relaxed atomistically.

For an overview over all performed CG and atomisitic simulations see Tables 1 and 2, respectively.

**Table 1:** Overview over all performed coarse-grained simulations.

Coarse-grained systems	Number of simulations	Simulation length
**Dimerization set-ups**		
**POPC**	501	3 *μ*s
**POPC/10%Cholesterol**	501	6 *μ*s
**POPC/30%Cholesterol**	499	6 *μ*s
**Monomers**		
**POPC**	10	200 ns
**POPC/10%Cholesterol**	10	1 *μ*s
**POPC/30%Cholesterol**	10	200 ns
**DEPC**	10	200 ns
**GMO/6%Cholesterol**	10	200 ns
**Membranes**		
**POPC**	1	200 ns
**POPC/10%Cholesterol**	1	200 ns
**POPC/30%Cholesterol**	1	200 ns
**DEPC**	1	200 ns
**GMO/6%Cholesterol**	1	200 ns
**TM5,6/TM5,6 crystal dimer**		
**POPC**	1	3 *μ*s
**POPC/10%Cholesterol**	1	3 *μ*s
**POPC/30%Cholesterol**	1	3 *μ*s
**TM1/TM5-7 crystal dimer**		
**POPC**	1	3 *μ*s
**POPC/10%Cholesterol**	1	3 *μ*s
**POPC/30%Cholesterol**	1	3 *μ*s
**TM1/TM1 crystal dimer**		
**POPC**	1	3 *μ*s
**POPC/10%Cholesterol**	1	3 *μ*s
**POPC/30%Cholesterol**	1	3 *μ*s

**Table 2:** Overview over all performed atomistic simulations.

Atomistic systems	Number of simulations	Simulation length
**Monomers in POPC**		
**CHARMM36**	1	500 ns
**Amber14/Lipid14**	1	200 ns
**pure POPC membranes**		
**CHARMM36**	1	500 ns
**Amber14/Lipid14**	1	510 ns
**TM5,6/TM5,6 crystal dimer in pure POPC**		
**CHARMM36**	1	200 ns
**Amber14/Lipid14**	1	200 ns
**TM3,4/TM3,4 dimer in POPC/30%Cholesterol**		
**GROMOS54a7**	1	100 ns

### System preparation

The dimer crystal structure 3OE0 [17] was obtained from the Protein Data Bank and adjusted in the following way: First, all non-protein atoms, one monomer, and lysozyme (cocrystallizing agent) were removed. Then, the mutations of residues L125W and T240P, required for crystallization, were mutated back to represent the wild type protein. In the end, two missing intracellular loops were modelled by MODELLER [49] (The loop comprising K67, K68, L69, and R70 was not resolved in the crystal structure and in place of residues S229 and K230 lysozyme was present in the crystal structure.). The MODELLER output structure was energy-minimized using the GROMOS54a7 force field [50]. Because the termini were not resolved in the crystal structure, the final model contained 279 (25–303) of the 352 residues of the CXCR4 protein.

### Coarse-grained simulations

The DAFT procedure involved the following steps: First, atomistic structures of CXCR4 were converted to the Martini2.2 CG force field [47] using *martinize* [47]. The secondary and tertiary structure of the protein was assured to be stable by applying a RubberBand network for all backbone bead pairs within the distance of 0.9 nm and excluding the *i* − *i* + 1 and *i* − *i* + 2 pairs (which are connected by bonds and angles within the Martini force field). The RMSD of the TM helices of CXCR4 was as low as 1.5 Å (500 ns, CHARMM36 force field) or even 1.0 Å (Amber14sb/Lipid14) in atomistic simulations of CXCR4 (see below). Therefore, the application of structural restraints in the coarse-grained model is not expected to influence the results on receptor dimerization.

CG proteins were randomly rotated around the z-axis and placed in pairs with the minimum distance of circumscribed spheres of 3.5 nm into a rhombic box. In the next step a CG lipid bilayer [51], either pure POPC or mixed POPC/cholesterol with 9:1 or 7:3 ratio, and CG water [52] were added by *insane* [53, 54]. Those systems were then passed to *martinate* [55] where they underwent 500 steps of steepest-descent energy minimization and 10 ps of position restrained NVT simulation with a 2 fs time step. Furthermore, 100 ps of MD simulation in an NpT ensemble with a 20 fs time step were performed to heat the system to 310 K and adjust the pressure to 1 bar. Production runs succeeded in an NpT ensemble, where the temperature was kept at 310 K by applying the Berendsen thermostat [56] with a 1 ps time constant. The pressure was controlled in a semi-isotropical manner (xy and z were independent) to 1 bar using the Berendsen barostat [56] with a 3 ps time constant. The relative permittivity was set to be 15 and the electrostatic interactions were shifted to 0 between 0 and 1.2 nm. The van der Waals forces were described by the 12-6 Lennard-Jones potential that was shifted to zero between 0.9 and 1.2 nm. The integration time step was 20 fs and the movement of the center of mass of the system was removed linearly every 10 steps.

#### CG dimerization simulations

A typical dimerization setup contained two copies of CXCR4, a lipid membrane made either out of approx. 320 POPC, 375 POPC/40 cholesterols (POPC/10% cholesterol) or out of 255 POPC/110 cholesterols (POPC/30% cholesterol) surrounded by about 7,000 CG water molecules, corresponding to a ratio of about 1:20 lipid:CG water.

#### CG crystal dimer simulations

The three different crystal dimers were extracted from the crystal structures 3OE9 (TM5,6/TM5,6 and TM1/TM1 dimers) and 3OE8 (TM1/TM5-7 dimer) and the sequence (both length and mutations required for crystallization) was adjusted to equal the sequence used for the DAFT setup described above. The crystal dimer simulations were performed in all three membrane compositions of interest (build by *insane* [53, 54]) and the protein:lipid:water ratios as well as the simulation procedure were chosen similar to the DAFT dimerization simulations.

#### CG monomer simulations

The positioning and dynamics of single CXCR4 molecules in membranes of different thickness and composition, was addressed in assays of 10 simulations each of 200 ns length. Cholesterol binding hotspots on CXCR4 in a membrane were studied at a reduced cholesterol content (POPC/10% cholesterol) for 1 *μ*s. All single protein simulations included one CXCR4 receptor, about 250 lipids, and were solvated by approx. 4,300 CG water molecules.

#### CG pure bilayers

Similarly, for comparison of lipid diffusion and the bilayer thickness in an unperturbed membrane, pure membrane systems (260 POPC, 304 POPC/32 cholesterols (POPC/10% cholesterol), 214 POPC/92 cholesterols (POPC/30% cholesterol), 246 DEPC, and 365 GMO/22 cholesterols (GMO/6% cholesterol) each solvated by about 4,300 CG water molecules) were simulated for 200 ns each.

A summary of all coarse-grained simulations is given in Table 1.

### Atomistic simulations

Atomistic simulation systems were set up in Gromacs 5.0.4 [57] by backmapping relaxed coarse-grained systems to an atomistic resolution (either CHARMM36 [58, 59] or Amber14sb/ Lipid14 [60–62] force fields) using *backward* [48]. In case of crystal structure simulations the corresponding monomer or dimer crystal structures were fitted on the backmapped structures and energy minimized. Before production run simulations, 2 ns simulations were performed with position restraints applied on the protein. The final simulation runs were performed in the NpT ensemble, where the temperature was kept constant at 310 K using the v-rescale thermostate [63] with a coupling time constant of 0.5 ps. The pressure was controlled semiisotropically to stay at 1 bar. The center of mass motion was removed for the whole simulation system. The simulation of the TM3,4/TM3,4 dimer in POPC with 30% cholesterol content was prepared in GROMACS 4.6.5 using the GROMOS54a7 [50] force field. The particle mesh Ewald summation [64] was applied to compute the long-range electrostatics. For further simulation details see Pluhackova et al [65]. A summary of all atomistic simulations is given in Table 2.

### Analysis

#### Dimerization criterium

Two proteins were considered to be a dimer, if the interaction energy (sum of Lennard-Jones and Coulomb interaction energy) between their TM helices (defined in Fig 1) was below -50 kJ/mol.

#### Dimer binding free energy

**Fig 6:**
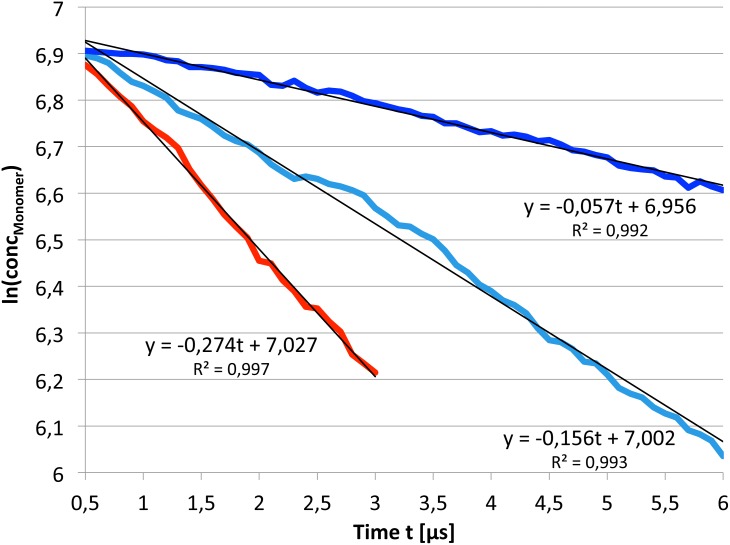
Concentration of receptor monomers as a function of simulation time. The first 500 ns were discarded for equilibration purposes.

Based on the time evolution of the number of dimerized simulations, the reaction rate constant *k* and the dissociation constant *K_D_* were estimated as follows: The dimerization reaction rate constant *k* was directly derived from the concentration of monomers in all simulations as a function of simulation time (Fig 6) assuming a first-order reaction. The rate constants decrease with the increased amount of cholesterol in the membrane (Table 3).

**Table 3:** Reaction rate constants, dissocation constants as well as binding free energies.

Setup	*k* in 10^6^ *s*^−1^ ^a^	*P_0_*/*P_1_* ^b^	*V* in *nm*^3^ ^c^	*K_D_* ^d^	Δ*G* in *kJ/mol* ^e^
**POPC**	0.274	0.0309	566.4	9.06 ⋅ 10^−5^	-23.98
**POPC/10%Cholesterol**	0.156	0.0858	610.2	2.34 ⋅ 10^−4^	-21.54
**POPC/30%Cholesterol**	0.057	0.1733	605.6	4.75 ⋅ 10^−4^	-19.71

*^a^* Dimerization reaction rate constants *k* derived from Fig 6.

*^b^* *P_0_*/*P_1_* denotes the ratio between times spent in monomeric states (after dissociation) and dimeric states in the simulation setups.

*^c^* *V* is the total volume of the protein-lipid bilayer.

*^d^* Estimated dissociation constants *K_D_*.

*^e^* The binding free energies Δ*G* should be considered as lower bounds for the true binding free energies as the number of dissociation events decreases with increased simulation times.

The dissociation constants *K_D_* and the corresponding binding free energies Δ*G* were approximated according to [66]:

$$K_{D}=\frac{P_{0}}{P_{1}c^{⊘}N_{Av}V}\;.$$

**Fig 7:**
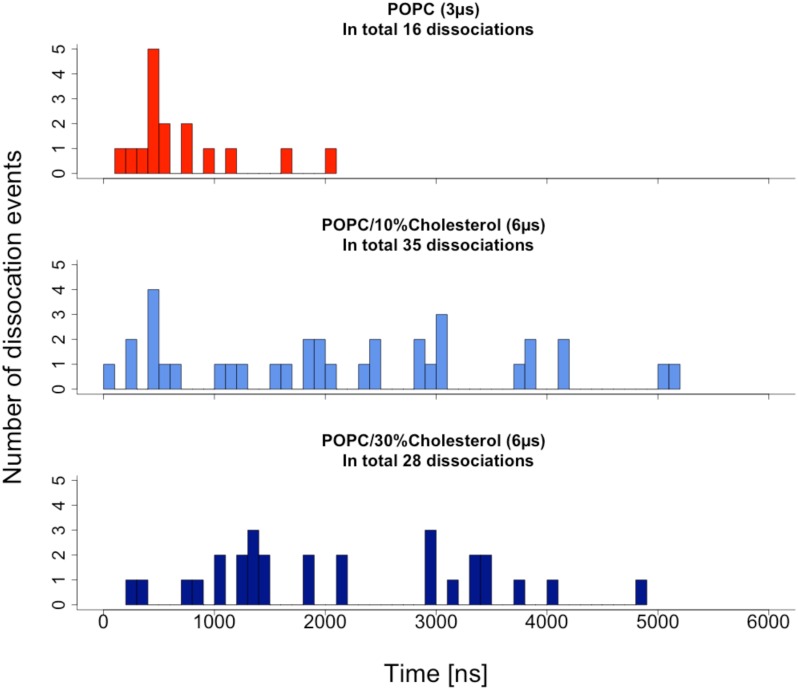
Histograms of monomer lifetimes after dissociation of dimers.

*c*^⊘^ is the standard concentration of 1 mol/l, *N_Av_* the Avogadro constant, and *V* the volume of the lipid-protein double layer. *P_0,1_* are the fractions of simulation time in monomeric and dimeric states, respectively. The above equation is valid in thermodynamic equilibrium; therefore, only those monomer states were counted that arose from dissociation of receptor dimers. Dissociation events were defined as a change of the protein-protein interaction energy from values below -50 kJ/mol to more than -1 kJ/mol. Histograms of these monomer lifetimes are shown in Fig 7 and the results for *K_D_* and Δ*G* = *RTlnK*_*D*_ are summarized in Table 3.

#### Orientation analysis

**Fig 8:**
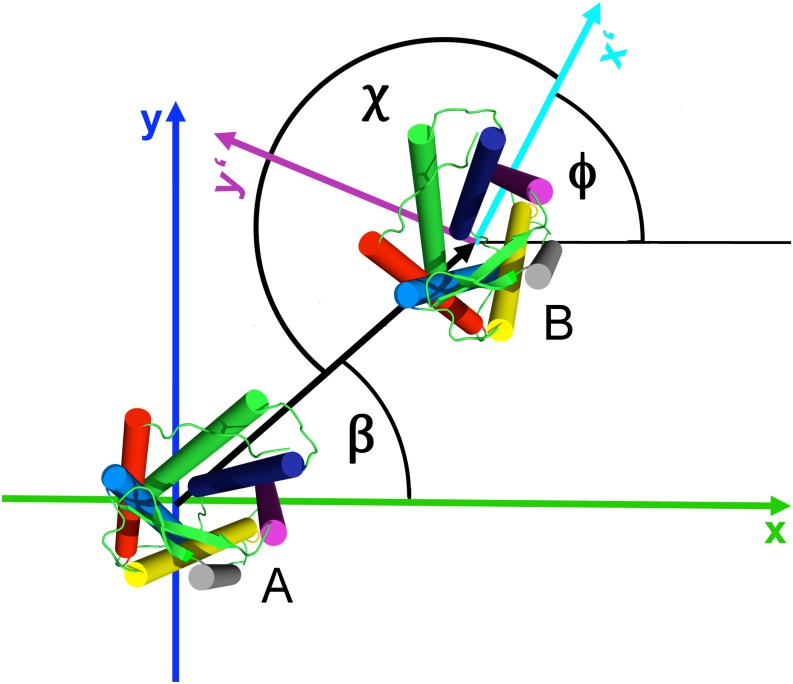
Relative orientation angles used to describe the orientation of the monomers in the receptor dimer. *β* describes the position of monomer B with respect to monomer A, *φ* is the rotation of monomer B around its z-axis, *χ* the angle under which monomer B “sees” monomer A. The axes x and y were placed into the monomer in a way that the symmetric TM5,6/TM5,6 dimer results in *β* = 0 and *χ* = 0.

Ensembles of simulations were analyzed to characterize the relative binding of the receptors. To that end, the configurations of the receptors were assigned a center of mass and principal components defining an internal coordinate frame (described in detail in [30]). From the superimposition of the coordinate frames, the COM distance and five angles were computed that describe the relative orientation of the proteins. The angles used in this work are graphically illustrated in Fig 8.

**Fig 9:**
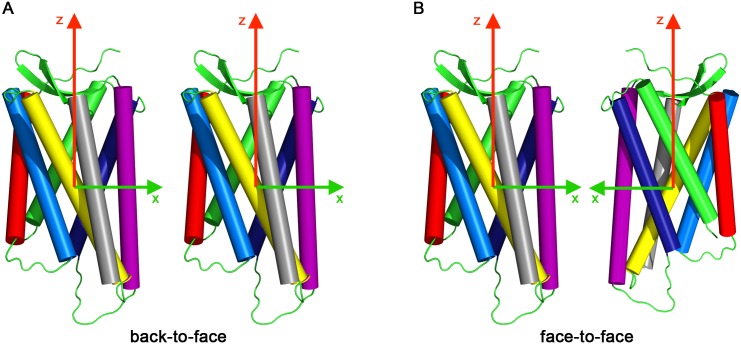
Relative orientation angles for TM5/TM1 or TM1/TM5 dimers a, corresponding to *β* = 0°, *χ* = 180° or *β* = 180°, *χ* = 0°, respectively. The symmetric TM5,6/TM5,6 dimer **b** binds with *β* = 0° and *χ* = 0°.

The position, angle *β*, defines the binding position of the partner to the reference protein. The rotation of the partner around its own z-axis is called phase and denoted as *φ*. The angle *χ*, calculated as *χ* = (180° + *β* − *φ*)*mod*360, describes under which angle the reference protein binds on the partner protein. Examples of two binding modes are shown in Fig 9. Panel *a* shows the so called back-to-face or also face-to-back binding mode corresponding to binding of helices TM5/TM1 or TM1/TM5 and angles *β* = 0°, *χ* = 180° or *β* = 180°, *χ* = 0°, respectively. In panel *b* the face-to-face binding of TM5,6/TM5,6 corresponding t o *β* = 0° and *χ* = 0° is shown. The *β* and *χ* angles were calculated for all spontaneously formed dimers for the last 50 ns of the simulation time. Subsequently, the kernel densities of all points in the *β*/*χ*-space were computed, which resulted in the density height-fields shown in Fig 2. Here the angles *β* and *χ* were sampled on the x and y axis, respectively. The probability of occurrences is the height encoded by color (white—red—yellow).

#### Finding label boundaries

The height-field resulting from the orientation angle analysis was examined more closely using methods from image processing.

First, the most frequent binding modes were found by computing the local maxima of the height-field. To examine the spreading of these maxima the watershed transform [67], a method originally used in image segmentation, of the height-field was computed. The basic idea is to consider the negative height-field as a topographic surface which is gradually filled with water and use the arising watersheds, where the water flows from one basin to another, as borders of the area of influence for the maximum assigned to each basin.

Simulated immersion [67] was adapted here to compute the watershed transform. Therefore, the pixels are sorted descending by height and a label image is initialized with distinct label values for each maximum. Subsequently, the 4-neighborhood of the sorted pixels was examined iteratively and the pixels were assigned to either one of the labels, if this label was unique in its neighborhood, or watershed, if two or more different labels were present. Pixels that could not be assigned in the first step, were processed in a second iteration where the neighborhood size was increased in case of slow convergence.

**Fig 10:**
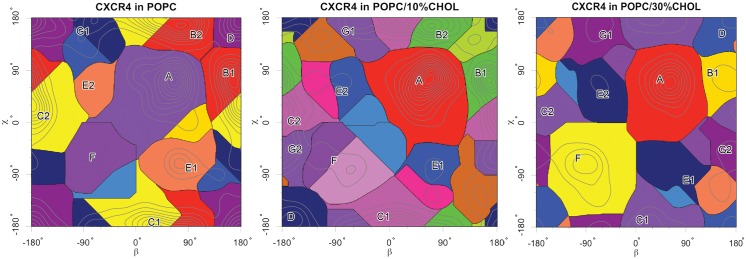
Spreading of each maximum in different dimerization set-ups.

As angles are circular data, the infinite repetition of the height-field in x and y direction had to be taken into account. By adding up the probabilities for all angle combinations falling into one area, the spreading of its maximum was computed. Results thereof are shown in Fig 10. In a next step, all simulation-frames were assigned to the different labels allowing for further differentiated analyses of dimer characteristics like the number of intercalating cholesterol molecules.

#### Lipid and protein lateral diffusion

Lateral self-diffusion coefficients of both lipids and proteins were calculated from the slope of the mean square displacement (MSD) of the beads averaged over the trajectories of each molecule, using the Einstein relation (see Table 4):

$$D_{s}=\lim_{t\to \infty }\frac{\left\langle \Delta r{\left(t\right)}^{2}\right\rangle }{4t}$$

where Δ*r*(*t*) is the distance that the bead travelled in time *t*. The slope of the MSD was fitted on the time window between 5–20 ns. The MSD was calculated on the initial 200 ns for those simulations that were not dimerized after 250 ns. The center of mass motion of the membrane-protein system was substracted prior to the MSD calculation.

**Table 4:** Diffusion constants.

Membrane	Molecule	*0*^a^	*1*^b^	*2*^c^
**POPC**	protein		0.44 ± 0.14	0.51 ± 0.13
	POPC	5.53	3.94 ± 0.21	3.67 ± 0.15
**POPC/10% cholesterol**	protein		0.33 ± 0.03	0.47 ± 0.12
	POPC	4.75	3.31 ± 0.06	3.26 ± 0.13
	Chol	6.23	4.15 ± 0.21	4.14 ± 0.31
**POPC/30% cholesterol**	protein		0.22 ± 0.07	0.26 ± 0.06
	POPC	2.78	2.12 ± 0.13	1.93 ± 0.08
	Chol	3.39	2.56 ± 0.10	2.26 ± 0.12

Protein and lipid diffusion coefficients in 10^−7^cm^2^/s (average and standard deviation).

*^a^* Pure bilayer simulations.

*^b^* Simulations including one copy of CXCR4 in the membrane.

*^c^* Simulations including two copies of CXCR4 in the membrane.

#### Cholesterol binding sites

A cholesterol molecule was considered as bound, if one of the 8 cholesterol beads was located within 0.62 nm of the protein. This distance corresponds to the size of the first solvation shell of cholesterol beads around the protein as obtained by the radial distribution function (RDF) calculation. The calculation was performed in an interval from 200 to 1000 ns for both cholesterol concentrations. The first 200 ns were discarded in order to exclude random protein-cholesterol contacts due to membrane construction around the proteins. In order to detect cholesterol binding residues, the average time for which any cholesterol was in contact with this residue was calculated. A similiar approach was used in [44]. Additionally, the number of cholesterol molecules located at the dimer interface in simulations with 10% and 30% cholesterol content was determined by analysis of cholesterol molecules bound to both monomers at the same time.

#### Bilayer thickness

The thickness (S1 Table) of phospholipid membranes was calculated as the difference of the maxima of the PO4 bead (or P atom in atomistic simulations) density profile along the membrane normal. In case of GMO, the density of its first bead (GL1) was utilized.

The bilayer thickness in the vicinity of the protein was calculated for lipids within 1 nm distance to the protein in each trajectory frame by substracting the z position of the PO4 bead (or GL1 bead for GMO or P atom in atomistic simulations) in the upper and lower leaflet by a home written script.

## Supporting Information

S1 Video Video of an example dimerization of CXCR4 in a pure POPC membrane. After approximately 1 *μ*s a stable dimer had assembled. The dimeric TM1/TM5-7 interface is shown in a coarse-grained representation first and later at atomistic resolution.
(MOV)

S2 Video Video of an example dimerization of CXCR4 in a POPC membrane with 30% cholesterol content. The dimer was formed after approximately 5 *μ*s. The symmetric TM3,4/TM3,4 dimer interface with intercalating cholesterol molecules is shown in a coarse-grained representation and is then backmapped into an atomistic model.
(MOV)

S1 Fig Evolution of number of dimers in time. Number of CXCR4 dimers formed in 500 simulations of two monomers each in phospholipid bilayers (POPC) at 0%, 10%, and at 30% cholesterol content as a function of simulation time. The receptors were defined to have formed a dimer if the intermolecular interaction energy between the two transmembrane domains decreased below a threshold of -50 kJ/mol.
(TIF)

S2 Fig TM5 tilt angles in coarse-grained simulations. Tilt angles between the first principal axis of the upper part (Asp192-Met205) or the lower part of TM5 (Val206-Ser277) and the membrane normal. For every environment (POPC, POPC/10% cholesterol, and POPC/30% cholesterol), the tilt angles of both monomers, shown for label *A* (TM5/TM5), *B* (TM1/TM5-7), and *D* (TM1/TM1), were calculated for the last 50 ns of the simulation and are shown in red. Angles of the best corresponding crystal dimer structures, calculated over the first 500 ns of CG simulations, are shown in blue (monomer A) and green (monomer B). In the inset, the tilt angles of receptors in monomer configuration are compared for different lipid environments of the receptor. The vertical lines denote the mean tilt angles as determined from the initial 500 ns of CG simulation of the given crystal CXCR4 dimers in pure POPC. Overall, the TM5 tilts are similar between monomer and the different spontaneously assembled dimers. These angles differ significantly from those for the TM5 orientation of simulations of the crystal symmetric TM5,6/TM5,6 dimer for all studied lipid compositions.
(TIF)

S3 Fig TM5 tilt angles in atomistic simulations. Comparison of tilt angles of upper (top) and lower (bottom) TM5 subhelices relative to the membrane normal in monomers and the TM5,6/TM5,6 dimer between coarse-grained and atomistic simulation. Overall, the TM5 tilt angles of the monomeric CXCR4 agree well between CG and atomistic simulations using the Amber14/Lipid14 force field combination. Atomistic simulations of the symmetric crystal dimer configuration exhibit asymmetric distributions for the tilt of the upper part of TM5, suggesting instability of this symmetry. The tilt angles of the lower part of TM5 show a very good agreement between CG and atomistic resolution using the Amber14/Lipid14 combination.
(TIF)

S4 Fig Spatial distribution function of cholesterol around CXCR4. The spatial distribution functions of the five nearest cholesterol molecules around CXCR4 are shown in light orange, distribution functions of the polar headgroup of cholesterol (ROH beads) are colored dark red. CXCR4 is colored according to the scheme established in Fig 1. The increasing cartoon thickness codes the residue-resolved cholesterol occupancy (compare Fig 4).
(TIF)

S5 Fig Cholesterol occupancy in POPC with 30% of cholesterol. Cholesterol binding occupancy in simulations of CXCR4 dimerization at 30% cholesterol concentration. The increasing cartoon thickness and coloring scheme code the relative cholesterol occupancies, from 0% (thin, blue) to 100% (thick, red).
(TIF)

S6 Fig Intercalation of cholesterol at dimer interfaces. Probability of finding at least one cholesterol molecule at different dimer interfaces for both mixed bilayers (top panel) and the number of intercalating cholesterol molecules (bottom panel). The analysis was performed over the final 50 ns of all simulations.
(TIF)

S1 Table Monomer tilt angles and membrane thickness. Tilt angles between the main principle axis of the protein’s transmembrane domain and the membrane normal, as well as membrane thicknesses.
*^a^* Total number of simulations in the respective set-up used for the analysis.
*^b^* Tilt angle of the principle axis of a CXCR4 monomer relative to the membrane normal (average and standard deviation).
*^c^* The thickness of the lipid bilayer in a 1 nm surrounding of the receptor.
*^d^* The thickness of the bilayer in protein-free simulations (one simulation per bilayer type and representation was performed). The CG membranes were simulated for 200 ns and the thickness was calculated over the last 100 ns.
*^e^* The simulation of a pure POPC bilayer using the (atomistic) CHARMM36 force field was carried out for 110 ns.
The preferred bilayer thickness around CXCR4 monomers in CG simulations was determined to ≈ 4.25 nm. Accordingly, CXCR4 locally thins membranes displaying a larger thickness in the simulations (POPC/30% cholesterol and 1,2-Dierucoyl-sn-glycerol-3-phosphocholine (DEPC) membrane), and increases the thickness of thin membranes (POPC). Interestingly, the thickness of a glycerol monoleate (GMO) environment for CXCR4 monomers is not significantly influenced by the presence of the receptor. The probable reason for this behavior is the missing charged phosphocholine headgroup of GMO. It is interesting to note, that CXCR4 was crystallized in a GMO/10% cholesterol PEG stabilized matrix. POPC membranes in atomistic simulations were found to be 2–3 Å thinner as compared to their CG counterparts. Accordingly, the thickness of atomistic POPC membranes was as well enlarged in the vicinity of the protein (at most for the Lipid14 parameters by 3 Å). The TM5,6/TM5,6 crystal dimer was observed to significantly thin both POPC and POPC/10% cholesterol membranes.
(PDF)

S2 Table Interconversions between different dimer interfaces. Comparison of the number of initially formed and final CXCR4 dimer configurations in pure POPC, POPC at 10% cholesterol, and POPC at 30% cholesterol content. Configurations marked by ‘*n*’ did not belong to any of the seven defined dimer configurations (labels *A* through *G*). Additionally, the total number of respective dimer configurations at the end of the microsecond association simulations is provided. In most of the simulations, the initially formed dimer configurations were stable, i.e. remained unchanged on the microsecond timescale.
(PDF)
