# Supplementary figures and images for: Dynamic Cholesterol-Conditioned Dimerization of the G Protein Coupled Chemokine Receptor Type 4
Source: PLoS Comput Biol. 2016 Nov 3;12(11):e1005169. doi: 10.1371/journal.pcbi.1005169 (PMC5094716; doi:10.1371/journal.pcbi.1005169)

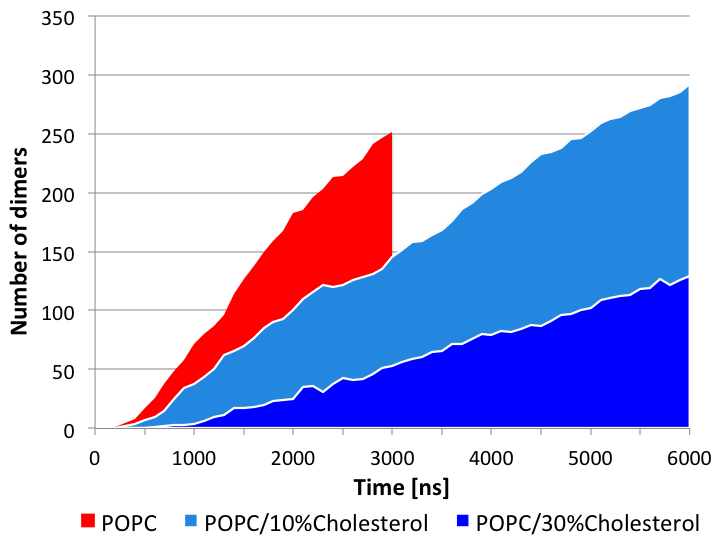

Supplement: S1 Fig — Number of CXCR4 dimers formed in 500 simulations of two monomers each in phospholipid bilayers (POPC) at 0%, 10%, and at 30% cholesterol content as a function of simulation time. The receptors were defined to have formed a dimer if the intermolecular interaction energy between the two transmembrane domains decreased below a threshold of -50 kJ/mol. (TIF) [file pcbi.1005169.s003.tif]

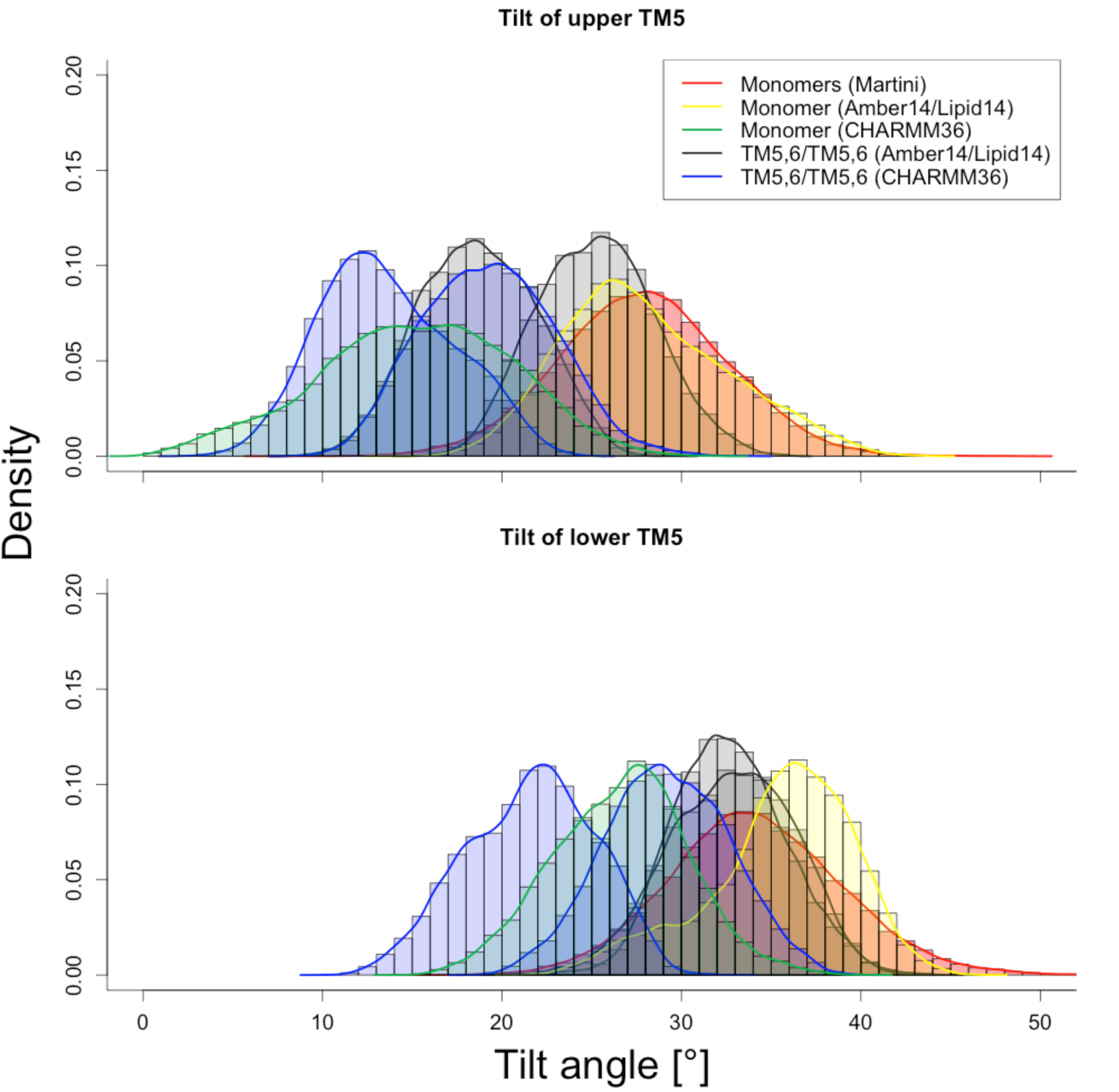

Supplement: S3 Fig — Comparison of tilt angles of upper (top) and lower (bottom) TM5 subhelices relative to the membrane normal in monomers and the TM5,6/TM5,6 dimer between coarse-grained and atomistic simulation. Overall, the TM5 tilt angles of the monomeric CXCR4 agree well between CG and atomistic simulations using the Amber14/Lipid14 force field combination. Atomistic simulations of the symmetric crystal dimer configuration exhibit asymmetric distributions for the tilt of the upper part of TM5, suggesting instability of this symmetry. The tilt angles of the lower part of TM5 show a very good agreement between CG and atomistic resolution using the Amber14/Lipid14 combination. (TIF) [file pcbi.1005169.s005.tif]

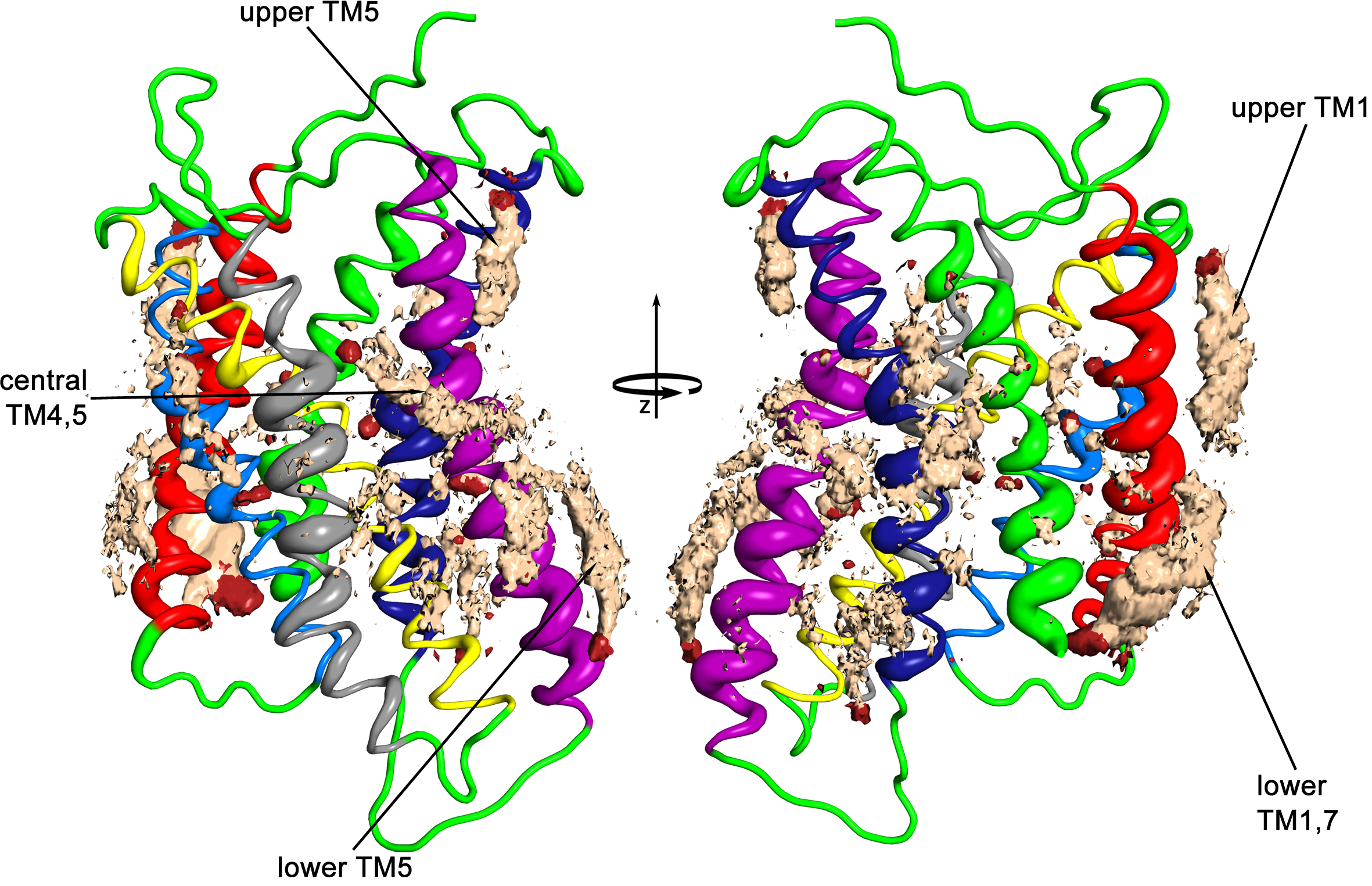

Supplement: S4 Fig — The spatial distribution functions of the five nearest cholesterol molecules around CXCR4 are shown in light orange, distribution functions of the polar headgroup of cholesterol (ROH beads) are colored dark red. CXCR4 is colored according to the scheme established in Fig 1. The increasing cartoon thickness codes the residue-resolved cholesterol occupancy (compare Fig 4). (TIF) [file pcbi.1005169.s006.tiff]

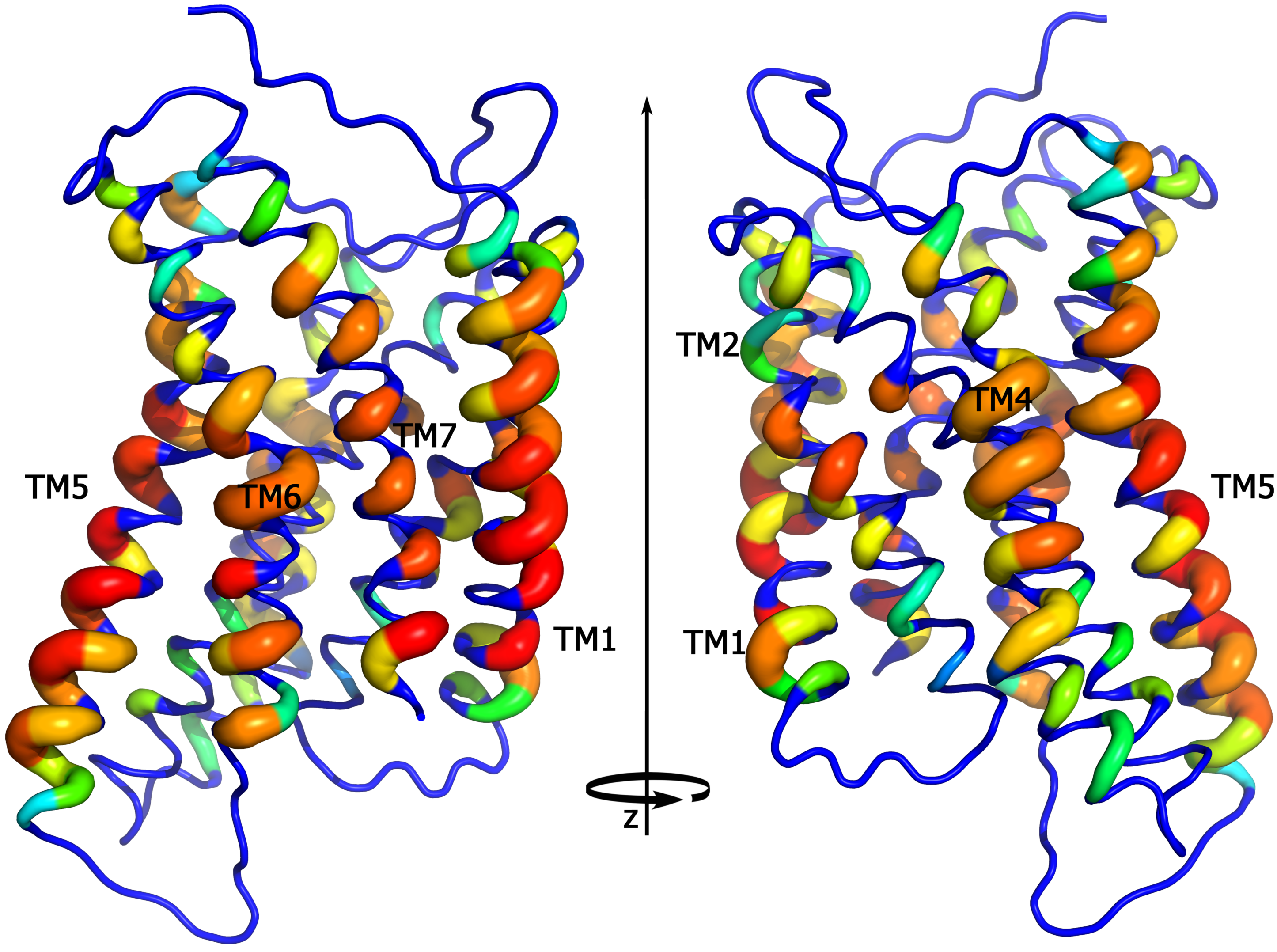

Supplement: S5 Fig — Cholesterol binding occupancy in simulations of CXCR4 dimerization at 30% cholesterol concentration. The increasing cartoon thickness and coloring scheme code the relative cholesterol occupancies, from 0% (thin, blue) to 100% (thick, red). (TIF) [file pcbi.1005169.s007.tif]

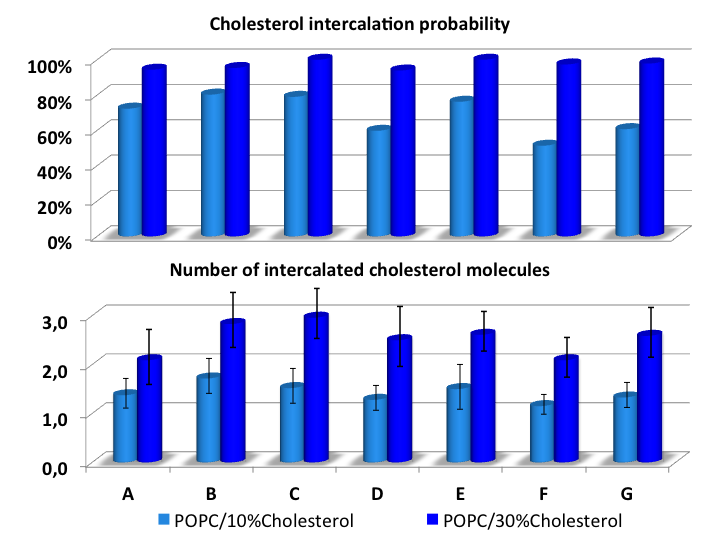

Supplement: S6 Fig — Probability of finding at least one cholesterol molecule at different dimer interfaces for both mixed bilayers (top panel) and the number of intercalating cholesterol molecules (bottom panel). The analysis was performed over the final 50 ns of all simulations. (TIF) [file pcbi.1005169.s008.tif]
