# Supplementary material for: Dynamic Cholesterol-Conditioned Dimerization of the G Protein Coupled Chemokine Receptor Type 4
Source: PLoS Comput Biol. 2016 Nov 3;12(11):e1005169. doi: 10.1371/journal.pcbi.1005169 (PMC5094716; doi:10.1371/journal.pcbi.1005169)
Supplement: S1 Table — Tilt angles between the main principle axis of the protein’s transmembrane domain and the membrane normal, as well as membrane thicknesses. a Total number of simulations in the respective set-up used for the analysis. b Tilt angle of the principle axis of a CXCR4 monomer relative to the membrane normal (average and standard deviation). c The thickness of the lipid bilayer in a 1 nm surrounding of the receptor. d The thickness of the bilayer in protein-free simulations (one simulation per bilayer type and representation was performed). The CG membranes were simulated for 200 ns and the thickness was calculated over the last 100 ns. e The simulation of a pure POPC bilayer using the (atomistic) CHARMM36 force field was carried out for 110 ns. The preferred bilayer thickness around CXCR4 monomers in CG simulations was determined to ≈ 4.25 nm. Accordingly, CXCR4 locally thins membranes displaying a larger thickness in the simulations (POPC/30% cholesterol and 1,2-Dierucoyl-sn-glycerol-3-phosphocholine (DEPC) membrane), and increases the thickness of thin membranes (POPC). Interestingly, the thickness of a glycerol monoleate (GMO) environment for CXCR4 monomers is not significantly influenced by the presence of the receptor. The probable reason for this behavior is the missing charged phosphocholine headgroup of GMO. It is interesting to note, that CXCR4 was crystallized in a GMO/10% cholesterol PEG stabilized matrix. POPC membranes in atomistic simulations were found to be 2–3 Å thinner as compared to their CG counterparts. Accordingly, the thickness of atomistic POPC membranes was as well enlarged in the vicinity of the protein (at most for the Lipid14 parameters by 3 Å). The TM5,6/TM5,6 crystal dimer was observed to significantly thin both POPC and POPC/10% cholesterol membranes. (PDF) [file pcbi.1005169.s009.pdf]

S1 Table Monomer tilt angles and membrane thickness.

System	N[a]	Analyzed time	Monomer [b] tilt [°]	d_P^c $[nm]$	d_M^d $[nm]$
**Dimerization setups (CG)**			**Monomers**		
**POPC**	184	2.95-3 μs	13.74±1.66	4.21	4.15
**POPC/10%Chol**	154	5.95-6 μs	13.45±1.95	4.24	4.26
**POPC/30%Chol**	312	5.95-6 μs	13.90±2.30	4.32	4.38
**Other membranes (CG)**			**Monomers**		
**DEPC**	10	150-200 ns	13.39±1.11	4.73	5.06
**GMO/6%Chol**	10	150-200 ns	15.59±2.10	3.54	3.55
**Atomistic setups (AA)**			**Monomers in POPC**		
**CHARMM36**	1	0-500 ns	28.73±5.55	4.08	3.96[e]
**Amber14/Lipid14**	1	0-200 ns	11.64±2.95	4.06	3.85
**Crystal dimers (CG)**			**TM1/TM1**		
**POPC**	1	0-50 ns	12.75±2.05	4.20	4.15
**POPC/10%Chol**	1	0-50 ns	10.75±2.23	4.25	4.26
**POPC/30%Chol**	1	0-50 ns	16.84±0.25	4.29	4.38
**Crystal dimers (CG)**			**TM1/TM5-7**		
**POPC**	1	0-50 ns	16.19±0.37	4.16	4.15
**POPC/10%Chol**	1	0-50 ns	17.88±1.01	4.18	4.26
**POPC/30%Chol**	1	0-50 ns	16.06±1.92	4.26	4.38
**Crystal dimers (CG)**			**TM5,6/TM5,6**		
**POPC**	1	0-50 ns	16.88±1.96	4.08	4.15
**POPC/10%Chol**	1	0-50 ns	16.42±0.54	4.06	4.26
**POPC/30%Chol**	1	0-50 ns	15.16±0.80	4.21	4.38
**Crystal dimers (AA)**			**TM5,6/TM5,6 in POPC**		
**CHARMM36**	1	0-200 ns	18.91±2.71	4.10	3.96
**Amber14/Lipid14**	1	0-200 ns	18.29±2.85	4.01	3.85

Tilt angles between the main principle axis of the protein's transmembrane domain and the membrane normal, as well as membrane thicknesses.

[a]Total number of simulations in the respective set-up used for the analysis.

[b]Tilt angle of the principle axis of a CXCR4 monomer relative to the membrane normal (average and standard deviation).

[c]The thickness of the lipid bilayer in a 1 nm surrounding of the receptor.

[d]The thickness of the bilayer in protein-free simulations (one simulation per bilayer type and representation was performed). The CG membranes were simulated for 200 ns and the thickness was calculated over the last 100 ns.

[e]The simulation of a pure POPC bilayer using the (atomistic) CHARMM36 force field was carried out for 110 ns. The preferred bilayer thickness around CXCR4 monomers in CG simulations was determined to $\approx$ 4.25 nm. Accordingly, CXCR4 locally thins membranes displaying a larger thickness in the simulations (POPC/30% cholesterol and 1,2-Dierucoyl-sn-glycerol-3-phosphocholine (DEPC) membrane), and increases the thickness of thin membranes (POPC). Interestingly, the thickness of a glycerol monoleate (GMO) environment for CXCR4 monomers is not significantly influenced by the presence of the receptor. The probable reason for this behavior is the missing charged phosphocholine headgroup of GMO. It is interesting to note, that CXCR4 was crystallized in a GMO/10% cholesterol PEG stabilized matrix. POPC membranes in atomistic simulations were found to be 2-3 Å thinner as compared to their CG counterparts. Accordingly, the thickness of atomistic POPC membranes was as well enlarged in the vicinity of the protein (at most for the Lipid14 parameters by 3 Å)The TM5,6/TM5,6 crystal dimer was observed to significantly thin both POPC and POPC/10% cholesterol membranes.
