# Supplementary material for: Dynamic Cholesterol-Conditioned Dimerization of the G Protein Coupled Chemokine Receptor Type 4
Source: PLoS Comput Biol. 2016 Nov 3;12(11):e1005169. doi: 10.1371/journal.pcbi.1005169 (PMC5094716; doi:10.1371/journal.pcbi.1005169)
Supplement: S2 Table — Comparison of the number of initially formed and final CXCR4 dimer configurations in pure POPC, POPC at 10% cholesterol, and POPC at 30% cholesterol content. Configurations marked by ‘n’ did not belong to any of the seven defined dimer configurations (labels A through G). Additionally, the total number of respective dimer configurations at the end of the microsecond association simulations is provided. In most of the simulations, the initially formed dimer configurations were stable, i.e. remained unchanged on the microsecond timescale. (PDF) [file pcbi.1005169.s010.pdf]

S2 Table Interconversions between different dimer interfaces.

Final interface	First interface		
	**POPC** (3 μs)	**POPC/10%Cholesterol** (6 μs)	**POPC/30%Cholesterol** (6 μs)
***A* (TM5,6/TM5,6)**	96.15% (*A*)<br>1.92% (*B*)<br>1.92% (*n*)<br>Total: 52	96.30% (*A*)<br>3.70% (*E*)<br>Total: 81	97.37% (*A*)<br>2.63% (*F*)<br>Total: 38
***B* (TM1/TM5-7)**	1.56% (*A*)<br>58.48% (*B*)<br>38.45% (*C*)<br>1.52% (*D*)<br>Total: 65	73.34% (*B*)<br>26.74% (*C*)<br>1.92% (*n*)<br>Total: 47	57.14% (*B*)<br>42.86% (*C*)<br>Total: 7
***C* (TM1/TM5)**	9.72% (*B*)<br>87.04% (*C*)<br>1.72% (*F*)<br>Total: 62	9.43% (*B*)<br>85.71% (*C*)<br>4.86% (*F*)<br>Total: 60	12.50% (*B*)<br>62.50% (*C*)<br>12.50% (*G*)<br>Total: 19
***D* (TM1/TM1)**	2.86% (*B*)<br>91.43% (*D*)<br>5.71% (n)<br>Total: 35	41.18% (*D*)<br>58.82% (*n*)<br>Total: 17	85.71% (*D*)<br>14.29% (*n*)<br>Total: 7
***E* (TM4/TM5-7)**	75.00% (*E*)<br>25.00% (*n*)<br>Total: 12	83.33% (*E*)<br>16.67% (*n*)<br>Total: 7	100.00% (*E*)<br>Total: 4
***F* (TM3,4/TM3,4)**	100.00% (*F*)<br>Total: 3	90.00% (*F*)<br>10.00% (*G*)<br>Total: 14	100.00% (*F*)<br>Total: 25
***G* (TM1,7/TM3-5)**	6.25% (*C*)<br>56.25% (*G*)<br>25.00% (*n*)<br>Total: 12	3.57% (*A*)<br>6.70% (*C*)<br>29.91% (*F*)<br>36.61% (*G*)<br>23.21% (*n*)<br>Total: 30	6.25% (*F*)<br>87.50% (*G*)<br>6.25% (*n*)<br>Total: 14

Comparison of the number of initially formed and final CXCR4 dimer configurations in pure POPC, POPC at 10% cholesterol, and POPC at 30% cholesterol content. Configurations marked by '*n*' did not belong to any of the seven defined dimer configurations (labels *A* through *G*). Additionally, the total number of respective dimer configurations at the end of the microsecond association simulations is provided. In most of the simulations, the initially formed dimer configurations were stable, i.e. remained unchanged on the microsecond timescale.
